# Exploring Hypotheses of the Actions of TGF-β1 in Epidermal Wound Healing Using a 3D Computational Multiscale Model of the Human Epidermis

**DOI:** 10.1371/journal.pone.0008515

**Published:** 2009-12-31

**Authors:** Tao Sun, Salem Adra, Rod Smallwood, Mike Holcombe, Sheila MacNeil

**Affiliations:** 1 Centre for Cell Engineering, University of Glasgow, Glasgow, United Kingdom; 2 Department of Computer Science, University of Sheffield, Sheffield, United Kingdom; 3 Department of Engineering Materials, University of Sheffield, Sheffield, United Kingdom; Cedars-Sinai Medical Center and University of California Los Angeles, United States of America

## Abstract

In vivo and in vitro studies give a paradoxical picture of the actions of the key regulatory factor TGF-β1 in epidermal wound healing with it stimulating migration of keratinocytes but also inhibiting their proliferation. To try to reconcile these into an easily visualized 3D model of wound healing amenable for experimentation by cell biologists, a multiscale model of the formation of a 3D skin epithelium was established with TGF-β1 literature–derived rule sets and equations embedded within it. At the cellular level, an agent-based bottom-up model that focuses on individual interacting units (keratinocytes) was used. This was based on literature-derived rules governing keratinocyte behavior and keratinocyte/ECM interactions. The selection of these rule sets is described in detail in this paper. The agent-based model was then linked with a subcellular model of TGF-β1 production and its action on keratinocytes simulated with a complex pathway simulator. This multiscale model can be run at a cellular level only or at a combined cellular/subcellular level. It was then initially challenged (by wounding) to investigate the behavior of keratinocytes in wound healing at the cellular level. To investigate the possible actions of TGF-β1, several hypotheses were then explored by deliberately manipulating some of these rule sets at subcellular levels. This exercise readily eliminated some hypotheses and identified a sequence of spatial-temporal actions of TGF-β1 for normal successful wound healing in an easy-to-follow 3D model. We suggest this multiscale model offers a valuable, easy-to-visualize aid to our understanding of the actions of this key regulator in wound healing, and provides a model that can now be used to explore pathologies of wound healing.

## Introduction

There is a wealth of intracellular detail available on the genome, proteome and metabolome of individual cells. Integration of this growing body of new data into a tissue biology can be aided by the use of *in virtuo* modeling [**1**]–[**2**]. Computational modeling can be used to organize complex biological data, connecting experimental results to fundamental biological principles. Such models can then be used to explore the role of a single parameter in a complex biological system, something that is rarely possible using *in vitro* or *in vivo* experimentation. Their major contribution to cell and tissue biologists however is that they aid and challenge our thinking about how parameters relate to each other, providing a relatively simple platform in which to test hypotheses and ultimately improve our understanding of complex biological systems such as tissue morphogenesis and pathogenesis [**1**]–[**4**].

Agent-based modeling in particular is popular with biologists as it can be used to simulate the interactions of autonomous entities (agents or cells) with each other and their local environment to predict higher level emergent behaviours. The outputs of these models can be visual and easily accessible to biologists to assist in developing the much needed interaction between cell and tissue biologists and computational modelers [**5**].

The underpinning hypothesis behind agent based modeling for cells is that the development of a complex tissue is crucially dependent on the coordination of relatively few cellular mechanisms [**1**], [**6**]. Thus in our previous work in this area we used a rule set which describes several basic cellular behaviors of keratinocytes (proliferation, migration and differentiation etc.) derived from the keratinocyte literature by abstracting the details of complex sub-cellular mechanisms to develop an agent-based colony formation model. This model was then used to generate some predictions which were tested in parallel *in vitro* experiments which allowed us to explore hypotheses about how normal human keratinocyte (NHK) form colonies [**5**].

In this study, we have progressed this work in two directions. We now model keratinocyte organization in three dimensions (3D) which allows us to simulate the formation of the epidermis (and then the response of the epidermis to wounding) and we have taken on a multi-scale modeling approach which allows us to link intracellular signaling rules (concerning one key growth factor, transforming growth factor beta1 (TGF-β1)) and the emergent behaviours of NHK at the cellular level.

Since the majority of the biological data of TGF-β1 are either qualitative or semi-quantitative, our general approach, as before, was to derive rule sets and/or relatively simple equations for TGF-β1 regulation of keratinocyte behaviour from the extensive literature on TGF-β1, which was then simulated with COmplex PAthway Simulator (COPASI, http://www.COPASI.org) [**7**] and linked to the agent based model. There are a growing number of models of intracellular regulation of the cell cycle or of the activity of particular key intracellular signaling molecules. We selected TGF-β1 to develop a multi-scale model because it is a potent tissue regulator with profound but apparently paradoxical influences on epithelial cell behaviour in wound healing. Clearly TGF-β1 is not the only regulator of epithelial cell behaviour but to the best of our knowledge this is one of only a few such multi-scale models which seek to link intracellular signaling to an agent based model of cell behaviour (as referenced in the accompanying paper).

The rule sets for the COPASI model were based on detailed analysis of the biological data describing TGF-β1 synthesis, expression, secretion, activation, signaling and biological functions during re-epithelialisation. Rule sets were deliberately designed so they could be readily modified to incorporate more quantitative biological data as this emerges in the future. The COPASI model was then integrated into the agent based re-epithelialisation model. Thus the behaviour of each cell agent was governed not only by rule sets at the cellular level, but also by TGF-β1 sub-cellular mechanisms that were simulated with COPASI. The assumptions used in the TGF-β1 simulations and in the cell level simulations and the relationships between the two are described in the Materials and Methods section.

The model was initially developed to describe the behaviour of keratinocytes and the expression and regulation of TGF-β1 following the wounding and healing of an epithelium. Then the model was used in exploratory *in virtuo* experiments to investigate the extent of the influence of TGF-β1 on epidermal wound healing. This *in virtuo* research demonstrated that the proliferative and migratory NHKs were located at different areas of the wound bed and both are necessary for normal epidermal wound healing. The model suggests that TGF-β1 plays a crucial role in normal re-epithelialisation by dictating the balance between cell proliferation and migration.

## Materials and Methods

The description of the model and how it was developed are described in the accompanying paper. This section however describes how the extensive literature on re-epithelialisation and TGF- β1 was selected and used to derive the rule sets in the model. The general approach was to conduct an extensive and systematic literature review for TGF- β1 and skin which encompassed normal adult skin, and its role in foetal development and adult wound healing using Medical Subject Headings (MeSH), “TGF-beta1” and “re-epithelialisation” and similar terms. Reports were further divided into the reported actions of TGF- β1 in cell division, migration and differentiation. Only those which were substantially confirmed in several laboratories were used in formulating the rule sets. Studies which differed from each other quantitatively but not qualitatively were viewed as essentially similar for the purposes of this initial rule set formulation. A representative selection of this literature is referred to beneath and was used for the *in silico* model.

### Review of Epidermal Wound Healing and the Broad Role of Cytokines Including TGF-β1

The normal human epidermis is a stratified squamous epithelium composed predominantly of keratinocytes at different stages of proliferation/differentiation [**8**]–[**10**]. Re-epithelialisation is the process of restoring an intact epidermis after cutaneous injury, which involves the migration, proliferation, stratification and differentiation of keratinocytes, and the gradual restoration of an intact basement membrane (BM) [**8**], [11]–[**13**].

The wounding of skin signals the transition of basal and supra-basal keratinocytes from a relative sedentary phenotype to migratory and hyperproliferative phenotypes [**9**]–[10], [13]–[**15**] and these activated keratinocytes do not proceed along their normal differentiation pathway but instead are directed (by a range of signals) to participate in the re-epithelialisation of the wound [**16**]. These signals include cytokines/growth factors, extracellular matrix (ECM), integrins, proteases (e.g. plasmin and matrix metalloproteinase (MMP)) and various other stimuli [**10**], [12], [14]–[15], [**17**].

Keratinocytes on the wound margins are constantly bombarded by signaling factors such as transforming growth factor *a* (TGF-*a*), TGF-β, activin, platelet-derived growth factor (PDGF), keratinocyte growth factor (KGF), epidermal growth factor (EGF) and heparin-binding EGF-like growth-factor (HB-EGF) etc [**9**]–[12], [18]–[**19**]. The different growth factors have varied but overlapping functions on the proliferation and differentiation of NHK [**20**]. Amongst these, TGF-β1 probably has the broadest range of activities [**11**], [21]–[**22**]. The reasons for selecting TGF-β1 to model in this study are twofold -it is acknowledged as a key influence in normal (and abnormal) wound healing and there is a wealth of experimental data on its expression and activities to draw on. In the re-epithelialisation model, the sub-cellular mechanisms of all growth factors were abstracted away (not modeled) when deriving the minimum rule set, but the details of TGF-β1's actions were simulated explicitly using COPASI.

### Modelling the Extracellular Matrix

The next aspect of wound healing to explain is the role and reconstitution of the basement membrane (BM)–a key structure of the extracellular matrix (ECM). ECM components have regulatory roles [**23**]–[**24**]. They can regulate the behaviours of keratinocytes directly [**25**] or indirectly by serving as a reservoir for various growth factors (e.g. fibronectin binds many growth factors) [**26**]–[**31**]. At different stages of re-epithelialisation, keratinocytes and other cells express different ECM components to restore the BM. Keratinocytes at the tip of the migration tongue synthesize and deposit ECM components such as laminin-5 precursor and fibronectin (Fn), and thus remodel the provisional matrix into a secondary matrix [**25**]. As re-epithelialisation ensues, they then express other proteins such as laminin to produce the basal lamina, and essentially remodel the secondary matrix into a BM in a sequential manner starting from the margins of the wound and moving inwards, in a zipper like fashion [**9**]–[10], [12], [**32**]. Meanwhile, these transient changes in the ECM induce remarkable changes in keratinocyte phenotype during re-epithelialisation [**33**]–[**35**]. For example, the presence of a provisional matrix signals the transition of basal keratinocytes from a sedentary to a migratory phenotype [**8**]–[10], [23]–[**24**], while the presence of a mature BM signals the transition of keratinocytes from a migratory to a sedentary phenotype [**9**]–[10], [12], [23], [**32**].

In this model, the ECM was simply divided into three types (provisional matrix, secondary matrix and mature BM) and simulated using three types of tile agents of 20 µm×20 µm respectively. The rules we used were that the provisional matrix indirectly promotes migration and inhibits proliferation of NHK by inducing the expression of TGF-β1 (simulated using COPASI at the sub-cellular level), the secondary matrix enhances NHK proliferation and migration and the mature BM inhibits NHK proliferation and migration (simulated using rules at the cellular level). Further the rules dictate that the interaction of NHK with the provisional matrix induces the remodeling of provisional matrix into secondary matrix, while the interaction of NHK with secondary matrix induces the remodeling of this matrix into BM (simulated using rules at the cellular level). In the integrated model, the dynamic interactions between NHK and the various ECM components were simulated using both biological rules at the cellular level and the COPASI model at the sub-cellular level, however the COPASI model was given a higher priority than the cellular rules.

### Review of Keratinocyte Migration

Keratinocytes migrate by a combination of keratinocyte integrin expression and remodelling of the underlying ECM by keratinocyte protease expression. In keratinocyte migration, the basal cells and some of the suprabasal cells at the wound margin begin to express integrins (e.g. α5β1, αvβ6, αvβ5) and relocalize α2β1 in order to grasp hold of, and crawl over, the provisional matrix. It has been reported that the expression of integrins that enable ‘motility’ extends more than 10 cells back from the leading edge, as well as upwards into cell layers above the basal layer in the advancing epidermal tip [**8**]–[10], [15]–[16], [24], [36]–[**37**]. To aid keratinocyte migration, the expressions of various proteases, such as MMP-1 are also up-regulated through cell-matrix interactions to specifically degrade ECM proteins such as native collagens [**9**]. The need for cells to express integrins and proteases is thought to explain the lag time between wounding and the initiation of epithelial migration [**8**]–[10], [24], [**37**]. Both provisional matrix and TGF-β1 induced expression of integrins and proteases and their influences on NHK migration were modelled explicitly using COPASI and integrated into the re-epithelialisation model.

Additionally other injury signals are also reported to regulate the behaviors of NHK during wound healing such as “free edge” effects, mechanical, chemical (e.g. Ca2+ and ATP) and electrical signals [**10**], [12], [15], [35]–[**36**]. These stimuli were not modelled explicitly in this research-see later for modelling of cell migration.

### Review of TGF-β1 Activity and Our Simulation Approach

In the normal human epidermis, relatively low levels of TGF-β1 are expressed predominantly in suprabasal, differentiating layers, suggesting it has a role in maintaining the cessation of growth in the differentiating cells of epidermis [**23**], [**32**]. During re-epithelialisation, the expression of TGF-β1 is induced by various ECM components. For example, the disruption of BM can dramatically enhance TGF-β1 promoter activity, TGF-β1 mRNA levels and thus the expression of latent TGF-β1 in keratinocytes, which can be further up-regulated by active TGF-β1 itself [**23**]. Meanwhile, TGF-β1 induces the secretion of various ECM proteins in an autocrine manner, contributing to the establishment of more physiological cell-ECM interactions. This subsequently down-regulates the expression of TGF-β1 through a feedback loop mechanism which maintains the balance between ECM remodelling and TGF-β1 synthesis [**8**], [23], [**38**]. The expression of TGF-β1 is thus confined within certain areas of the wound bed [**8**], [23], [**38**].

In this integrated model, stem cells and or Transit Amplifying (TA) cells express TGF-β1 when (1) they are stratified and a certain distance away from the matrix surface, (2) in contact with a provisional matrix, (3) under the regulation of TGF-β1, which can be down regulated by the presence of a secondary matrix and BM components. In this model all of these TGF-β1 expression regulation signals are detected by cell agents through interrogation of the message lists and passed to the TGF-β1 COPASI model. The subsequently induced sub-cellular mechanisms from the activation of TGF-β1 promoter activity and induction of mRNA to the synthesis of TGF-β1 large latent complex (LLC) are simulated by COPASI. The promoter activity level (PRO) depends on the presence of a provisional matrix (PM), secondary matrix (SM), active TGF-β1 ligand receptor complex (LRC) on the cell membrane and the cell stratification distance (D). The equations describing this are shown in the accompanying paper. All these equations and the coefficient factors used in this study were defined to reflect current biological research based on careful data mining. (These equations can be modified as desired to reflect for example new quantitive findings as these emerge or to explore the role of TGF-β1 in abnormal wound healing).

The expression of LLCs was simulated for 30 mins for each iteration and the synthesised LLCs were directly deposited onto the matrix underneath the cells or onto the membrane on neighbouring cells (as discussed in the next sections). The highest production of LLCs from each cell was set as 200millimolar/30 mins. The life cycles of active and latent TGF-β1 were modifiable parameters and both were set as 48 hours, that is, both types of TGF-β1 could degrade within 48 hours [**32**].

In order to explain how TGF-β1 reaches the appropriate target cell in biologically relevant concentrations at the correct time, various explanations have been employed to oppose entropy, augment the process of diffusion and concentrate and store TGF-β1 in the ECM [**21**]–[22], [26]–[31], [38]–[**39**]. Firstly, TGF-β1 is synthesized as biologically inactive large latent complexes (LLCs) composed of a latent TGF-β binding protein (LTBP) covalently bound to the latency associated protein (LAP) and TGF-β. Because of the covalent association between LTBP and specific ECM components such as fibronectin (Fn), most of the secreted LLCs are concentrated and fixed in ECM [**23**], [26]–[**31**].

The subsequent retrieval of latent TGF-β from the ECM and its activation is a critical regulatory step in the action of TGF-β1 [**22**]–[23], [27], [38], [40]–[**41**]. There are two main biological mechanisms to release active TGF-β1: (1) conformational change of LLCs [**40**], [**42**] by its direct interaction with cell surface receptors or proteins such as integrins (e.g. αvβ1, αvβ8, αvβ6) and thrombospondin (TSP)-1; (2) proteolysis of LAP by proteases such as plasmin and MMP [**30**], [**40**]. During wound healing, the αvβ6 induced TGF-β1 liberation and activation from the ECM plays an important role [**37**], [40], [**42**], which requires close associations among αvβ6, LAP, LTBP and Fn [**21**], [23], [26]–[31], [37], [**40**]. Both TGF-β1 and Fn are parts of a feed forward loop regulating ECM formation and TGF-β1 activation, since Fn plays important roles in TGF-β1 storage and activation, meanwhile TGF-β1 induces the synthesis and incorporation of Fn into the ECM. This feed forward mechanism is organized mainly by activating cells at the time of TGF-β activation within a confined area [**30**], [**37**] as both TGF-β expression and activation can also be suppressed by a remodelled ECM [**21**], [**43**]. Consequently, the deposition and activation of TGF-β1 are also spatially restricted within certain areas by several mechanisms [**8**], [23], [**38**]. A notable exception to the coordinated activation process for immobilised TGF-β1 is that it is released in abundance in its active form by platelets and macrophages at the site of injury [**8**], [22], [27], [38], [**44**].

As the above literature analysis shows, a random diffusion equation could not simulate the process of TGF-β1 concentration and fixation into the ECM. Thus the amount of latent TGF-β1 deposited into the ECM was simply simulated to be directly related to the expression level of TGF-β1, which was taken to reflect the specific concentration and fixation mechanisms. In provisional matrix more latent TGF-β1 can be deposited due to the presence of ECM proteins such as Fn, but less latent TGF-β1 can be deposited in a secondary matrix and in mature BM due to the remodelling of ECM components. The LLC in the matrix was only activated by the cells that directly contacted with the ECM matrix and the activated TGF-β1 on the cell membrane was simulated to be directly related to the level of TGF-β1 in the ECM to reflect the αvβ6 induced specific activation mechanism on the membrane.

### Modelling TGF-β1 Regulation of Cellular Processes

TGF-β1 binds to three high-affinity cell-surface receptors known as types I, II, and III (TbRI, II, III). TbRIII functions by binding and transferring TGF-β1 to TbRII, but TGF-β1 can bind to TbRIIs with or without the help of TbRIIIs. Once activated by TGF-β1, TbRIIs recruit, bind and transphosphorylate TbRIs. The TbRI then activates the downstream effectors (i.e. Smad2 and Smad3) by phosphorylation. The activated Smad proteins form complexes with the common Smad mediator, Smad4, and then translocate to the nucleus, where the Smad complexes regulate transcription of TGF-β1 target genes in conjunction with various transcriptional or co-transcriptional regulators [**19**], [21]–[22], [**45**]. Other non-Smad signaling pathways can also be activated by TGF-β in a context-dependent manner [**39**], [**45**].

A peculiarity of the TGF-β pathway is that receptors are constitutively internalized, even in the absence of ligand. The different kinetics of biosynthesis, degradation and trafficking of TbRI and TbRII can modulate TGF-β signalling [**21**], [**46**] and these have already been simulated using COPASI [**47**].

In the present study a recently published TGF-β signalling COPASI model [**47**], was used as part of our own COPASI model. Using this the activated TGF-β1 on the cell membrane can be used by the COPASI model of TGF-β signalling [**47**], to simulate the level of ligand-receptor on the membrane of endosomes (LRE). This is then used by the “TGF-β function” COPASI model to simulate various function of TGF-β1 as discussed in the following sections.

TGF-β1 is a pleiotropic growth factor with both growth-promoting and growth-suppressive activities depending on circumstances, including dose, target cell type and context [**11**], [22]–[23], [39], [45], [48]–[**50**]. At first glance the role of TGF-β1 in the re-epithelialization process appears paradoxical. It is a strong inhibitor of keratinocyte proliferation [**39**], [**50**] and, it promotes cell migration by inducing the expression of integrins and proteases [**8**], [10]–[11], [19], [23], [51]–[**52**]. TGF-β1 also regulates the behaviour of keratinocytes by inducing the expression of various ECM proteins such as tenascin, thrombospondin, fibronectin, vitronectin, collagen and several proteoglycans [**8**], [21]–[23], [30], [45], [52]–[**53**]. (None of these are modelled explicitly in this study but rather this level of detail has been abstracted away into our simple model of the ECM).

These apparently contradictory functions of TGF-β1 on wound closure have been confirmed in several different biological models **[8]**, [11], [19], **[38]**. In this integrated model, the influence of TGF-β1 on the expression of integrins, proteases and the proliferation of keratinocytes depends on the level of ligand-receptor complexes in the endosome (LRE) as simulated explicitly using the “TGF-β1 function” COPASI model as discussed in the following sections.

### Development of the Agent and COPASI-Based Integrated Model

Based on the extensive published data on epidermal wound healing and TGF-β1 biology (as described above), we developed an agent and COPASI based integrated model. Biological rules of the emergent behavior of NHKs induced by injury were derived and incorporated into the previously developed keratinocyte colony formation model to establish an agent based re-epithelialisation model. TGF-β1 expression, signaling and function were then simulated using COPASI and integrated within the re-epithelialisation model. Most of the biological rules used in the previous keratinocyte colony formation model [**5**] were kept un-changed except for slight alterations to simulate cell behavior in the human epidermis in 3D as opposed to in 2D culture. Briefly, NHK stem cells can attach to the surface of ECM, proliferate, form tight colonies, and control the size of the stem cell colony automatically. When the stem cell colony reaches a certain size, the stem cells on the colony edge will differentiate to TA cells. TA cells can migrate, divide, stratify and control the size of TA cell colony due to the auto-regulation mechanisms. When TA cells are a certain distance away from stem cells, they will differentiate to committed cells. Committed cells gradually lose their nuclei and further differentiate to corneocytes [**5**].

All the new biological rules, the modifications of the original rules and the simulation of TGF-β1 in the integrated model are described in the following sections. Moreover, in addition to the biological rules and the TGF-β1 intra-cellular functions modeled with COPASI, a simple numerical based physical model constituting a biomechanical layer was deployed to resolve the forces exerted between cells which mainly result from cell migrations and mitosis. The physical solver deployed is a 3D version of the solver used previously [**54**] and was responsible for correcting some anomalies such as cell overlapping resulting from mitosis by applying repulsive forces which are proportional to the amount of overlap. The physical solver was also responsible for cell stratification and resolving attractive forces.

#### 1. Modelling of cell migration

Within hours after the wounding of adult skin, keratinocytes at the wound margin start to flatten, elongate, develop pseudopod-like projections of lamellipodia and migrate toward the denuded area [**12**]–[13], [**55**]. The essential mechanisms responsible for the motility or flux of cells including epithelial cells are mitotic activity, cell active movement, cell-cell and cell-substrate interactions [**56**]. In the re-epithelialisation model, there are attractive and repelling forces between different agents as in the previous model. The attractive forces simulate cell-cell and cell-substrate bonds, and are applied when the respective bodies (cell-cell or cell-substrate) are within 5 µm of one another, which keeps keratinocytes in the coherent cell sheet and the epidermis to the surface of ECM. In the integrated model, however, the cell-cell and cell-substrate bonds were simulated using variables instead of constants, which can be regulated by the function pathway of the TGF-β1 COPASI model. The ligand-receptor level in the endosome (LRE) simulated by the TGF-β1 signaling pathway in COPASI was used by the TGF-β1 function pathway to further simulate the expression of integrins and proteases (IN_PR) according to the equations described in the accompanying paper. Based on the expression of integrins and proteases (IN_PR), the cell-cell bond (CCB) and cell-substrate bond (CSB) will be modified by the physical model according to the equations described in the accompanying paper.

Epidermal cells generally maintain cell-cell contacts and migrate as a coherent sheet, rather than as single free entities [**8**], [15]–[16], [35]–[**36**]. Moreover, keratinocytes in the normal wound healing process are regulated to migrate toward the denuded area on the provisional matrix in response to specific cytokines and ECM proteins. For example, keratinocytes in the migrating front deposit laminin 5, which serves as a track to allow subsequent keratinocytes to migrate on [**55**]. Thus two alterations were made to the active migration of TA cells as used in our previous model. Firstly, TA cells can migrate actively at the same rate (1 µm/minute) as in the previous model provided they keep contact with other cell agents. Secondly, a high tendency for the TA cells to migrate toward or on the provisional matrix is defined for active migration of TA cells.

#### 2. Modelling of cell proliferation

Although epidermal cells are able to control their cell number due to auto-regulation mechanisms [**3**], [33]–[**34**], environmental factors such as a range of injury signals also play an important role in regulating the proliferation of basal cells. From about 12 hours to 1–2 days after wounding and some hours after the onset of migration, there is a marked increase in mitotic activity in the basal cells a small distance away from the wound edges [**9**]–[10], [**12**], providing an extra source of basal cells for the supplement of the advancing and migrating epithelial tongue [**55**]. In addition to the auto-regulation mechanism, the influences of the ECM matrix and TGF-β1 on cell proliferation were simulated explicitly, while the influences of other factors (such as cytokines) on cell proliferation were modeled implicitly in the re-epithelialisation model. For example it is well accepted that cells in a wound bed will become more proliferative and also migrate further away from the centre of their original colonies because of a high local concentration of cytokines. In order to simulate this hyperproliferation of the cells in the wound bed, a slight modification was made to the biological rule set so that both stem and TA cell agents could migrate further away from the centre of stem or TA cell colonies before they start to differentiate, thus the sizes of these auto-regulated stem and TA colonies were deliberately made bigger than in the previous model.

The cell proliferation rates of both stem and TA cells were also simulated using variables instead of constants, thus the cell proliferation rate can be regulated by the TGF-β1 function pathway of the COPASI model. Thus the ligand-receptor level in the endosome (LRE) simulated by the TGF-β1 signaling pathway is used by the TGF-β1 function pathway of the COPASI model to downregulate the proliferation rates (PRR) of stem and TA cells, which are also influenced by other factors such as the ECM components as discussed earlier.

#### 3. Modelling of cell differentiation

In this integrated model, the rule for differentiation was maintained between committed cells and corneocytes (as we previously published for our 2D model of keratinocyte colony formation) as *in vivo* there are gradients of many differentiation related signals. No attempt was made to model any of the pro-differentation factors in detail in this model-the rule used was that only the cells in the stratified layers could differentiate into committed cells and corneocytes. Cells once in this layer (now viewed as TA cells) differentiated as in our previous 2D model.

## Results

**Figure 1:**
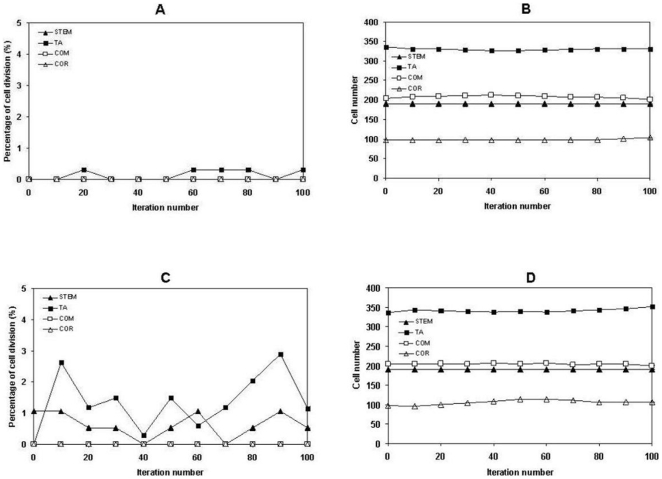
The influence of cell proliferation rate on homeostasis of the virtual epidermis. Under default proliferation rates (stem 1%, TA cell 2%), the intact virtual epidermal was simulated for 100 interactions, then (A) the actual cell proliferation rates and (B) the total numbers of the cells at different layer of the virtual epidermis were monitored. The proliferation rates were then increased 10 times higher (stem 10%, TA cell 20%), the intact virtual epidermal was also simulated for 100 interactions, then (C) the actual cell proliferation rates and (D) the total numbers of the cells at different layer of the virtual epidermis were monitored.

1. Exploration of the regulation of epithelial formation using the integrated model. Homeostasis is one of the main properties of the epidermis so *in virtuo* experiments were first carried out to test whether the intact *in virtuo* epidermis, which was created with the agent and COPASI integrated model in the accompanying paper had this property even when challenged by changing the initial proportion of the cells that were proliferative. Firstly, the intact virtual epidermis was simulated for 100 iterations under default cell proliferation conditions (stem cell 1%, TA cell 2%). Cell proliferation was found mainly in the stem and TA cell compartments as expected (Fig. 1A) and the overall 3D structure of the stratified squamous epithelium was maintained as the total number of different types of NHKs remained at a very stable level (Fig. 1B).(This is explicable as terminally differentiated cells are lost from the model). Then the cell proliferation rate was deliberately increased to 10 times higher than the default level (stem cell 10%, TA cell 20%), and yet very similar results were obtained as shown in (Fig. 1C–D). This indicates the robustness of the model in terms of its capability to simulate the basic structure of an intact epidermis providing the inherent rules governing colony formation (and hence differentiation) are not altered.

**Figure 2:**
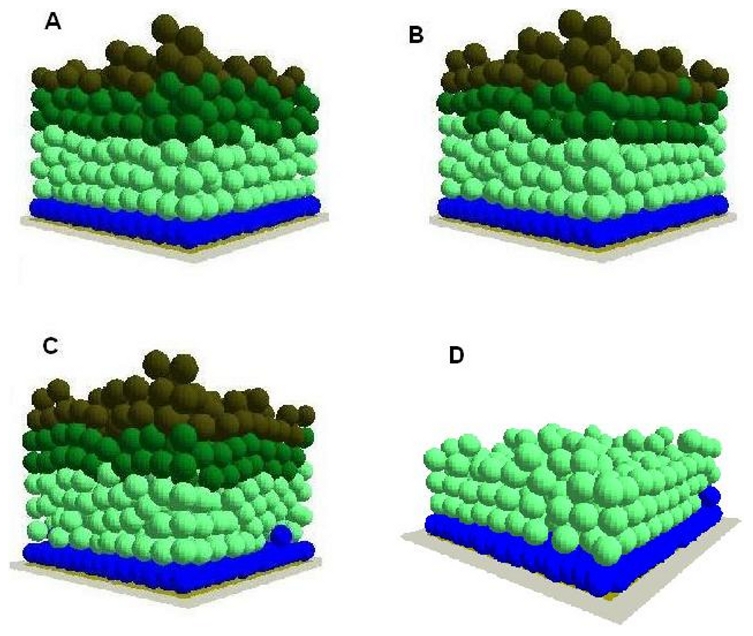
The influence of cell differentiation on homeostasis of the virtual epidermis. Under high proliferation rates (stem 10%, TA cell 20%), the differentiation rule in the agent based model was relaxed then the intact virtual epidermal was simulated for (A) 0, (B) 50,(C) 100 and (D) 100 interactions. However, the committed cells and corneocytes are not shown in (D). In the integrated model different colours were used to represent keratinocyte stem cells (blue), TA cells (light green), committed cells (dark green), corneocytes (brown), provisional matrix (dark red), secondary matrix (Green), BM tile agent (light purple). Cell agent diameter = 10 µm.

Then the differentiation rules in the model were relaxed and the intact virtual epidermis was simulated for 100 iterations under high cell proliferation (stem cell 10%, TA cell 20%) and default cell proliferation (stem cell 1%, TA cell 2%) conditions. It was found that under high cell proliferation conditions, some of the stem cells stratified but did not differentiate into TA cells (Fig. 2B–D), and similar results were observed in TA cells as the TA cell layer became thicker and some TA cells existed in the committed cell layer (Fig. 2C–D and Table 1) compared to the normal virtual epidermis, which begins to simulate some of the features of psoriasis. However, this was not obvious under default cell proliferation conditions even when the differentiation rules in the model were relaxed and the intact virtual epidermis was also simulated for 100 iterations (Table 1), suggesting psoriasis might be related to both cell proliferation and differentiation.

**Table 1:** The influences of cell proliferation and differentiation on homeostasis of the virtual epidermis.

Cell type	Default proliferation condition	Default proliferation condition Differentiation rules relaxed	High proliferation condition	High proliferation condition Differentiation rules relaxed
Stem cells	190	190	190	191
TA cells	330	335	**348**	**361**
Committed cells	202	200	202	190
Corneocytes	104	103	108	106

The total number of different types of keratinocytes were counted after intact virtual epidermal was firstly simulated for 100 interactions under default (stem 1%, TA cell 2%) and high proliferation rates (stem 10%, TA cell 20%), then the differentiation rule in the agent based model was relaxed and the intact virtual epidermal was simulated for 100 interactions under default (stem 1%, TA cell 2%) and high proliferation rates (stem 10%, TA cell 20%).

**Figure 3:**
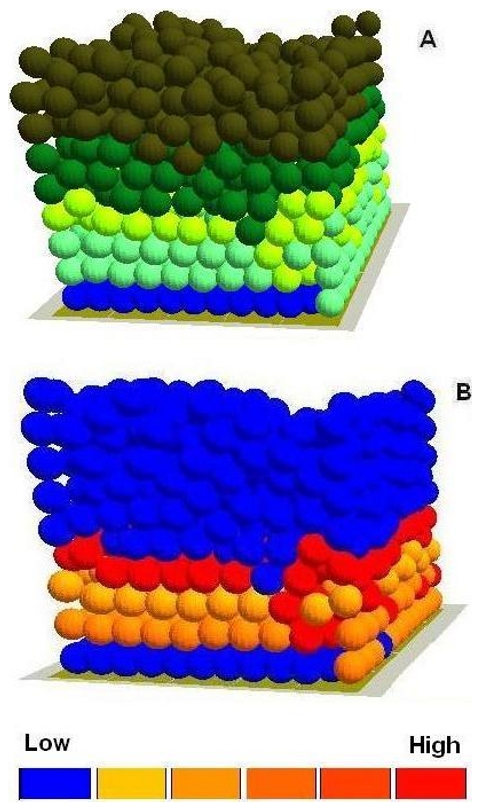
The different concentration levels of TGF-β1 in the virtual epidermis. In the virtual epidermis the stratified cells with relatively high expression level of TGF-β1 were labelled with yellow colour (A), In the integrated model different colors were used to represent keratinocyte stem cells (blue), TA cells (light green), committed cells (dark green), corneocytes (brown), provisional matrix (dark red), secondary matrix (Green), BM tile agent (light purple). Some of the cells with relative low expression level TGF-β1 were also illustrated using a simple thermal (B). Cell agent diameter = 10 µm. As an example, the sub-cellular parameters and the exact positions of the selected 9 cells in the epidermal wound were listed in Table 2.

2. TGF-β1 expression in the intact epidermis. In intact epidermis, the expression of TGF-β1 is said to be limited to cells with a certain level of differentiation [23], [32]. As Fig. 3 and Table 2 demonstrate, the expression of TGF-β1 in the intact virtual epidermis was also limited to such stratified TA cells - the further away from the BM, the higher the expression levels of TGF-β1 in these stratified TA cells, suggesting that this multi-scale model can simulate the *in vivo* situation.

**Table 2:** The stratification distances from the basement membrane and the sub-cellular parameters of 9 cells selected from the virtual epidermis as shown in Fig. 4.

Stratification distance (z)	Promoter level	mRNA level	TGF-Beta1	LRE level
11.58	0	0	10	0
16.98	0	0	10	0
10.03	0	0	10	0
28.91	0.75	11.35	130.21	12.37
20.57	0.74	11.21	118.53	11.99
21.29	0.77	11.60	125.35	12.25
30.10	0.78	11.65	135.37	12.57
32.46	0.84	12.55	150.85	13.16
37.08	0.94	14.04	176.64	14.15

Note: LRE level is the TGF-β1 ligand-receptor complex level on the membrane of endosome.

**Figure 4:**
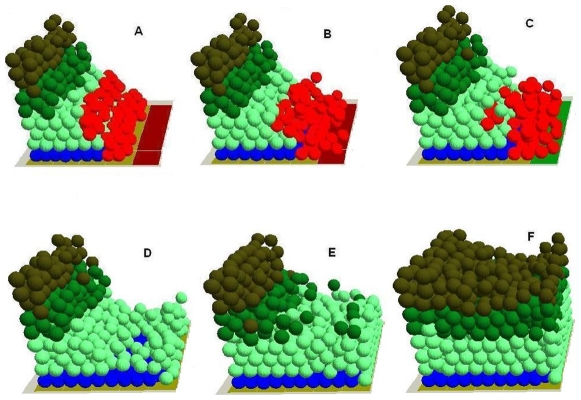
Simulation of the normal epidermal wound healing process using the ‘virtually injured’ epidermis. During the healing process, (A) some basal and superbasal cells (labeled with red colour) initially migrated from the wound margins onto the provisional matrix while proliferation/division of these cells was not observed. (B) as the epidermal cells on the wound surface migrated away, there was a burst of keratinocyte proliferation at the wound margins, thus two distinguishable NHK populations (migrative cells (red) and proliferative cells) appeared, (C) due to the cell-ECM interactions, the provisional matrix was remodelled into secondary matrix, (D) when totally covered the denuded area, basal keratinocytes started to stratify and differentiate into TA cells, the secondary matrix was gradually remodelled into basement membrane due to the cell-ECM interactions, (E) TA cells further stratified and differentiated into committed cells and then (F) corneocytes, and finally the virtual epidermal wound was totally re-epithelized. In the integrated model different colours were used to represent keratinocyte stem cells (blue), TA cells (light green), committed cells (dark green), corneocytes (brown), provisional matrix (dark red), secondary matrix (Green), BM tile agent (light purple). Cell agent diameter = 10 µm.

3. In virtuo investigation of epidermal wound healing. To create a virtual epidermal wound, the *in virtuo* intact epidermal model was deliberately ‘injured’ by removing all of the cells in the epidermis down to the BM. The underlying damaged dermis surface was then covered with provisional matrix ‘tiles’ to simulate the provisional matrix in the wound bed as one of the emergent events (Fig. 4A). To simulate the active TGF-β1 released by platelets and macrophages in the early stage of the wounding the TGF-β1 level at the wound surface was set to a high level. Running the model using the ‘virtually injured’ epidermis demonstrated that some basal and suprabasal cells (shown in red) initially migrated from the wound margins onto the provisional matrix while proliferation of these cells was not observed (Fig. 4A). Model analysis clearly indicated that active TGF-β1 (released by platelets and macrophages) [**8**], [22], [27], [38], [**44**] played an important role at the early stage of epidermal wound healing. In the presence of this active TGF-β1, the cells on the dermal wound bed surface were more motile and less proliferative. Thus the earliest stage of cell migration in this model was not driven by the relatively slow up-regulation of cell proliferation as reported [**8**]–[10], [24], [37], [55], [**57**]. Then as the epidermal cells on the wound surface migrated into the wound bed, there was a burst of keratinocyte proliferation at the wound margins, which provided the extra source of NHK for the continuous epidermal migration. As wound healing progressed, two distinguishable NHK populations appeared, proliferating keratinocytes mainly confined to the wound margins and migrating keratinocytes mostly distributed on the epithelial migration tongue moving toward the provisional matrix (Fig. 4B). This emergent behaviour of the model simulates results from several studies of TGF-β1 [**8**]–[10], [**58**].

When the basal keratinocytes had totally covered the denuded area they then started to stratify and differentiate into TA cells, which further stratified and differentiated into committed cells and then corneocytes, and finally the virtual epidermal wound was totally re-epithelialised (Fig. 4C–F). As re-epithelization progressed, a provisional matrix was gradually remodeled into a secondary matrix and then into a mature BM by the keratinocytes (Fig. 4B–D) [**9**]–[10], [12], [25], [32], [**55**]. During the re-epithelialisation process, all the epidermal cells were observed to migrate as a coherent sheet as reported [**8**], again showing that the model simulated the normal epidermal wound healing process.

**Figure 5:**
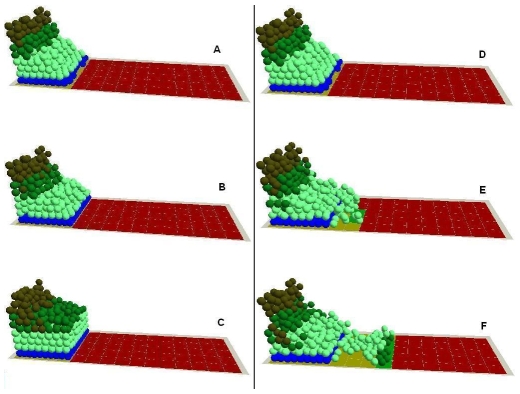
In virtuo investigation of the influence of cell proliferation and migration on epidermal wound healing. The migration rule at cellular level was completely blocked and the virtual epidermal wound was simulated for (A) 0, (B) 200, (C) 400 interactions. The hyperproliferation rule at cellular level was then blocked, and the virtual epidermal wound was simulated for (D) 0, (E) 200, (F) 400 interactions. In the integrated model different colours were used to represent keratinocyte stem cells (blue), TA cells (light green), committed cells (dark green), corneocytes (brown), provisional matrix (dark red), secondary matrix (Green), BM tile agent (light purple). Cell agent diameter = 10 µm.

**Figure 6:**
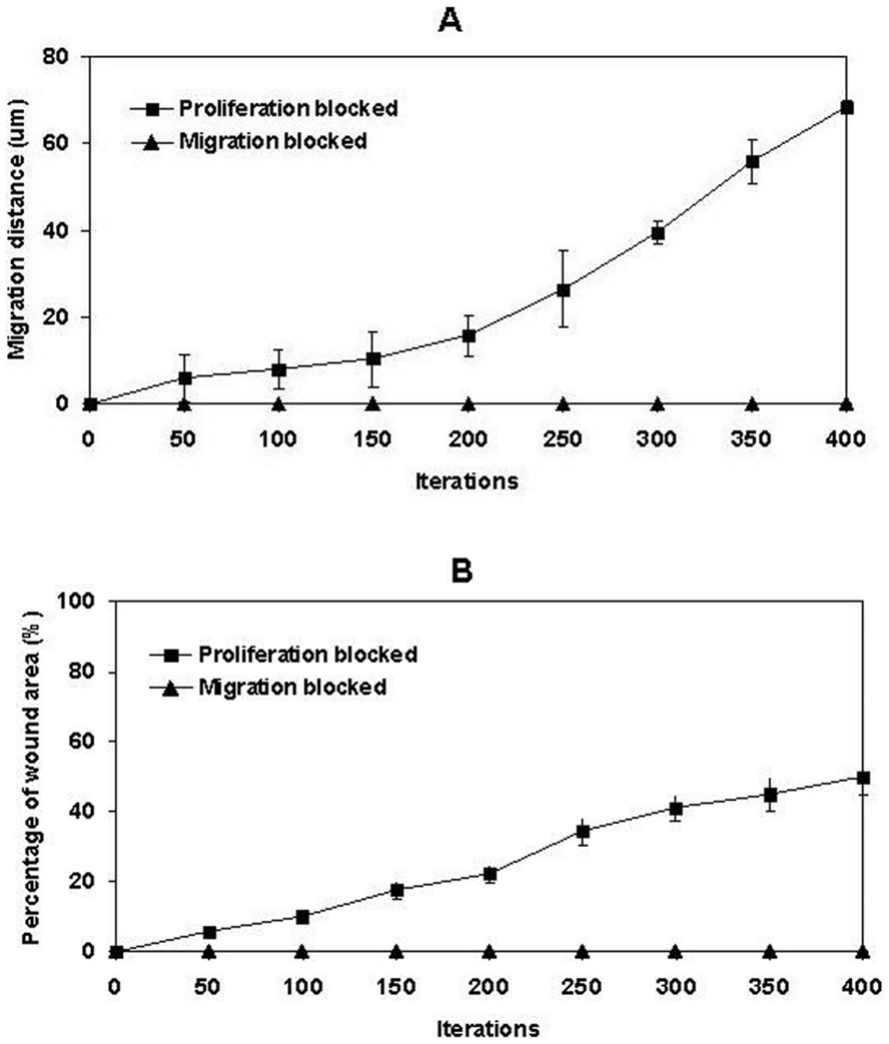
In virtuo investigation of the influence of cell proliferation and migration on epidermal wound healing. The migration and hyperproliferation rules at cellular level were blocked respectively in two in virtuo experiments and the virtual epidermal wound was then simulated 400 interactions. (A) Migration distance and (B) percentage of the wound area that covered by epidermal cells were used to evaluate the influences of the block of migration or hyperproliferation rules on epidermal wound healing.

4. Cell proliferation vs migration during epidermal wound healing. The comparative requirements for cell proliferation and migration in epidermal wound healing are not easy to determine. It has been suggested that re-epithelialisation does not appear to depend on cell proliferation since molecules such as TGF-β1 promote the migration of epithelial cells in organ cultures while acting as a potent inhibitor of keratinocyte proliferation, [**9**], [**59**]. Model analysis at the cellular level indicated that when the migration rule was completely blocked, wound healing failed as all the epithelial cells simply started to overlap on top of each other at the wound margin instead of migrating forward to cover the denuded area (Fig. 5A–C). When only the hyperproliferation rule was blocked, part of the wound healed at a relatively slow rate due to the low proliferation rate on the wound edges (Fig. 5D–F). However, when all the cell proliferation was inhibited, the wound healing process was completely deterred except for the small area near the wound edge which was covered by the initial cell migration of superbasal cells (data not shown). The influence of the block on migration rule or hyperproliferation rule on epidermal wound healing was also evaluated by measuring the migration distance of epidermal cells and the percentage of the wound area that was covered by cells as shown in Fig. 6A–B. Model analysis demonstrated that, at the earliest stage of re-epithelialisation, cell migration was not driven by cell proliferation as this was inhibited by factors such as TGF-β1 [**10**], [55], [**57**]. After all the dedifferentiated basal and superbasal cells migrated onto the denuded area, cell proliferation then became crucial for further progress of re-epithelialisation. The model showed that for a large wound re-epithelialisation is almost impossible without the presence of an extra source of new keratinocytes. Therefore, our *in virtuo* experimentation clearly demonstrated that both cell proliferation and migration are crucially important for re-epithelialisation–particularly for extensive wounds. One implication is that the failure to heal of chronic wounds may be caused by abnormal (or blocked) cell migration or a failure in cell proliferation or both.

**Figure 7:**
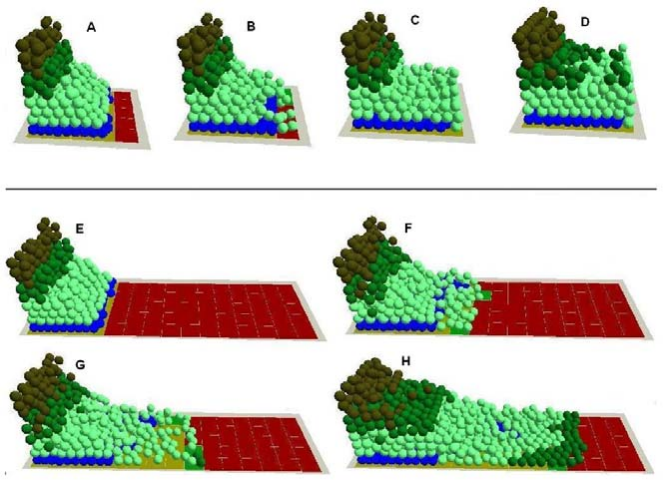
In virtuo investigation of the influence of the auto-regulation mechanism and time period on the healing processes of small and large wound beds. The small virtual wound with normal proliferation and migration rates were simulated for (A) 0, (B) 50, (C) 120, (D) 500 iterations and the epidermal cells migrated to cover the denuded area. The large virtual wound with normal proliferation and migration rates were simulated for (E) 0, (F) 200, (G) 400, (H) 800 iterations and keratinocytes on the wound bed started to differentiate into committed cells before the epidermal cells migrated to cover the large denuded area. In the integrated model different colours were used to represent keratinocyte stem cells (blue), TA cells (light green), committed cells (dark green), corneocytes (brown), provisional matrix (dark red), secondary matrix (Green), BM tile agent (light purple). Cell agent diameter = 10 µm.

The model was then used to investigate the re-epithelialisation process in a relatively large wound. The model predicted that even when cells have normal proliferation and migration rates, large wounds still cannot be healed no matter how long the model simulation is executed (Fig. 7E–H) compared with smaller wound beds (Fig. 7A–D). The healing process of a large wound using the integrated model is also illustrated in the attached Movie S1. Model analysis showed that this is because keratinocytes in this model are still regulated by the auto-regulation colony forming mechanism. Thus before the cells can migrate sufficiently to cover the large denuded area, they differentiate into committed cells and or corneocytes. Therefore, in such large wounds it will be necessary either to introduce a new source of keratinocytes not yet terminally differentiated (in clinical practice this would be achieved using skin grafts or tissue engineered skin) or to override the differentiation rules of these cells. The model as configured would achieve reepithelialisation of wounds of up a certain size (i.e. the size of stem and TA cell colony)–wounds greater than this would be predicted not to heal spontaneously.

In this research relatively small areas of epithelium were modelled simply to save simulation time, however, in clinical practice any full thickness burn wound of greater then 4 cm diameter would be considered to require skin grafting for healing. To make the *in virtuo* model dimensions closer to normal physiology this maximum size of a full thickness wound that can heal in practice could be introduced into the model to investigate parameters such as the colony sizes of stem and TA cells during wound healing, which are difficult to obtain in reality.

**Figure 8:**
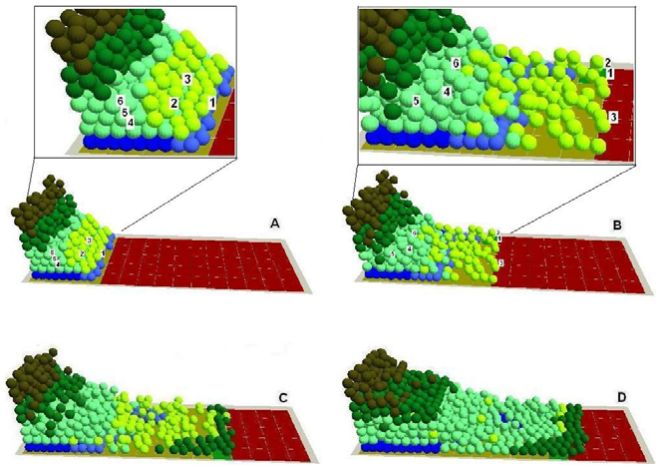
In virtuo investigation of the influence of TGF-β1 on epidermal wound healing at subcellular level. The virtual wound with normal proliferation and migration rates were simulated for (A) 0, (6 cells randomly selected as shown in the insert and their sub-cellular parameters and the exact positions were also in Table 3), (B) 200, (6 cells randomly selected as shown in the insert and their sub-cellular parameters and the exact positions were also in Table 4), (C) 400 and (D) 800 iterations. The cells with high TGF-β1 expression levels were labelled with yellow colour. In the integrated model different colours were used to represent keratinocyte stem cells (blue), TA cells (light green), committed cells (dark green), corneocytes (brown), provisional matrix (dark red), secondary matrix (Green), BM tile agent (light purple). Cell agent diameter = 10 µm.

5. In virtuo investigation of TGF-β1 signaling during epidermal wound healing. In the agent and COPASI based multi-scale model, both the cellular level behaviour and sub-cellular TGF-β1mechanisms were simulated and monitored. This allows us to now select a range of cells of interest during wound healing for detailed investigation at both the cellular and sub-cellular levels. Fig. 8A (insert) and Table 3 show the sub-cellular parameters and the exact positions of the 6 cells selected at an early stage of the wounding, while Fig. 8B (insert) and Table 4 show the sub-cellular parameters and the exact positions of these cells during wound healing. The influence of TGF-β1 on epidermal wound healing was then investigated at the sub-cellular level.

**Table 3:** The positions and the sub-cellular parameters and of the randomly selected 6 cells in the epidermal wound as shown in Fig. 8A.

Cell No.	Promoter level	mRNA level	TGF-Beta1	LRE level	Cell position		
					x	y	z
1	0	0	100	10	69.46	41.00	9.08
2	0	0	100	10	43.75	16.40	18.37
3	0	0	100	10	50.07	60.88	18.07
4	0	0	10	0	49.93	69.27	10.15
5	0	0	10	0	19.11	5.00	18.53
6	0	0	10	0	14.99	95.20	9.36

Note: LRE level is the TGF-β1 ligand-receptor complex level on the membrane of endosome.

**Table 4:** The positions and the sub-cellular parameters and of the randomly selected 6 cells in the epidermal wound as shown in Fig. 8B.

Cell No.	Promoter level	mRNA level	TGF-Beta1	LRE level	Cell position		
					x	y	z
1	1.49	22.40	314.09	19.83	136.71	72.62	0.48
2	1.05	15.68	198.00	15.38	131.73	94.10	0.91
3	1.32	19.86	270.24	18.15	139.72	34.19	0.96
4	0	0	10	0	40.72	59.78	27.40
5	0	0	10	0	31.02	29.60	18.22
6	0	0	10	0	28.14	70.93	9.15

Note: LRE level is the TGF-β1 ligand-receptor complex level on the membrane of endosome.

As shown in Fig. 8A, at the initial stage of wound healing, the active TGF-β1 released by platelets and macrophages at the site of injury initiated the outgrowth of epidermal cells by facilitating keratinocyte migration [**60**]. The cells regulated by TGF-β1 became less proliferative and were induced to express TGF-β1 (Fig. 8A). Consequently, the injury induced initial migration of basal and superbasal cells [**8**]–[10], [24], [**37**] was not driven by the relatively slow up-regulation of cell proliferation as discussed earlier [**10**], [55], [**57**]. After the epidermal cells migrated away and the initially deposited active TGF-β1 was gradually lost from the wound surface, the inhibitory effect of TGF-β1 on cell proliferation was also diminished and there was a subsequent burst of keratinocyte proliferation at the wound margins, which provided the extra source of NHK to maintain the progress of re-epithelialization (Fig. 8B–C).

As the epidermal cells migrated onto the provisional matrix, the synthesis, secretion, deposition and activation of TGF-β1 were gradually up-regulated due to these cell-matrix interactions. Interestingly, all of these events were confined to a certain proportion of cells located at the migrating epithelial tongue (Fig. 8B). Thus the cells in this area were promoted to be even more motile, but their proliferation was inhibited, while the proliferation of the keratinocytes beyond this area was not negatively influenced [**8**], [23], [**38**] (as shown in Fig. 8B–C). In addition to the influence of TGF-β1 synthesis, secretion, deposition and activation, there are other mechanisms to down-regulate the expression of TGF-β1 in the keratinocytes behind the migration tongue. Model analysis demonstrated that both the gradual restoration of BM and degradation of latent TGF-β1 in the matrix were the main mechanisms. As the influence of TGF-β1 diminished gradually, the cells behind the migration tongue started to resume proliferation, thus new cells were produced to maintain the continuous migration of the cells at the migration tip.

The model analysis clearly suggests that TGF-β1 provides a mechanism to coordinate the behaviour of proliferative and migratory keratinocyte populations, consequently the wound can be repaired in a coordinated and organised manner. The model also helps illustrate how the functions of TGF-β1 can be explained by its temporal-spatial distribution in normal wound healing process as described by several researchers [**8**], [**61**]. Consequently, overproduction, prolonged synthesis or malfunction of TGF-β1 might cause abnormal wound healing [**8**], [**62**].

In order to further investigate the influence of TGF-β1 on epidermal wound healing, its functions on cell migration and proliferation were then blocked and simulated in this model. In these *in virtuo* experiments the assumption that was made was that TGF-β1 was only responsible for 60% of stimulated cell migration and 60% of reduced cell proliferation, since it is only one of several growth factors that can regulate cell behaviour and there is no quantitative biological data suggesting to what extent this growth factor is responsible for cell migration and proliferation. (This figure of 60% is purely arbitrary and could easily be changed based on more accurate biological data).

**Figure 9:**
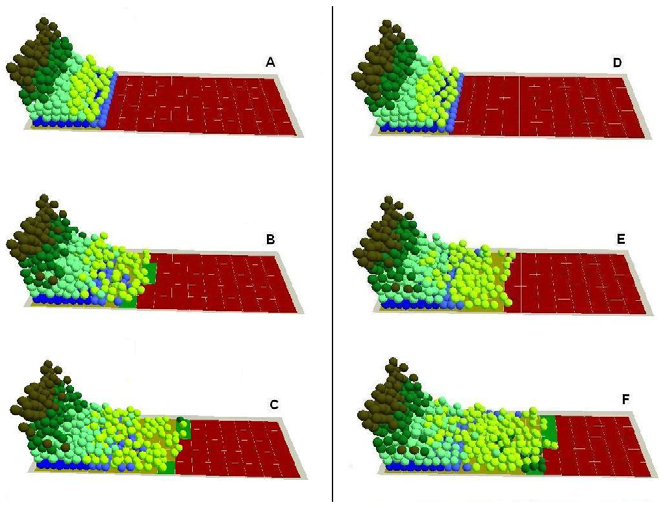
In virtuo investigation of the functions of TGF-β1 during epidermal wound healing. TGF-β1 was assumed to be responsible for 60% of cell migration and inhibit 60% of cell hyperproliferation. Its migration function was blocked and the virtual epidermal wound was simulated for (A) 0, (B) 200, (C) 400 interactions. In another in virtuo experiment, its hyperproliferation inhibition function was blocked, and the virtual epidermal wound was simulated for (D) 0, (E) 200, (F) 400 interactions. In the integrated model different colours were used to represent keratinocyte stem cells (blue), TA cells (light green), committed cells (dark green), corneocytes (brown), provisional matrix (dark red), secondary matrix (Green), BM tile agent (light purple). Cell agent diameter = 10 µm.

**Figure 10:**
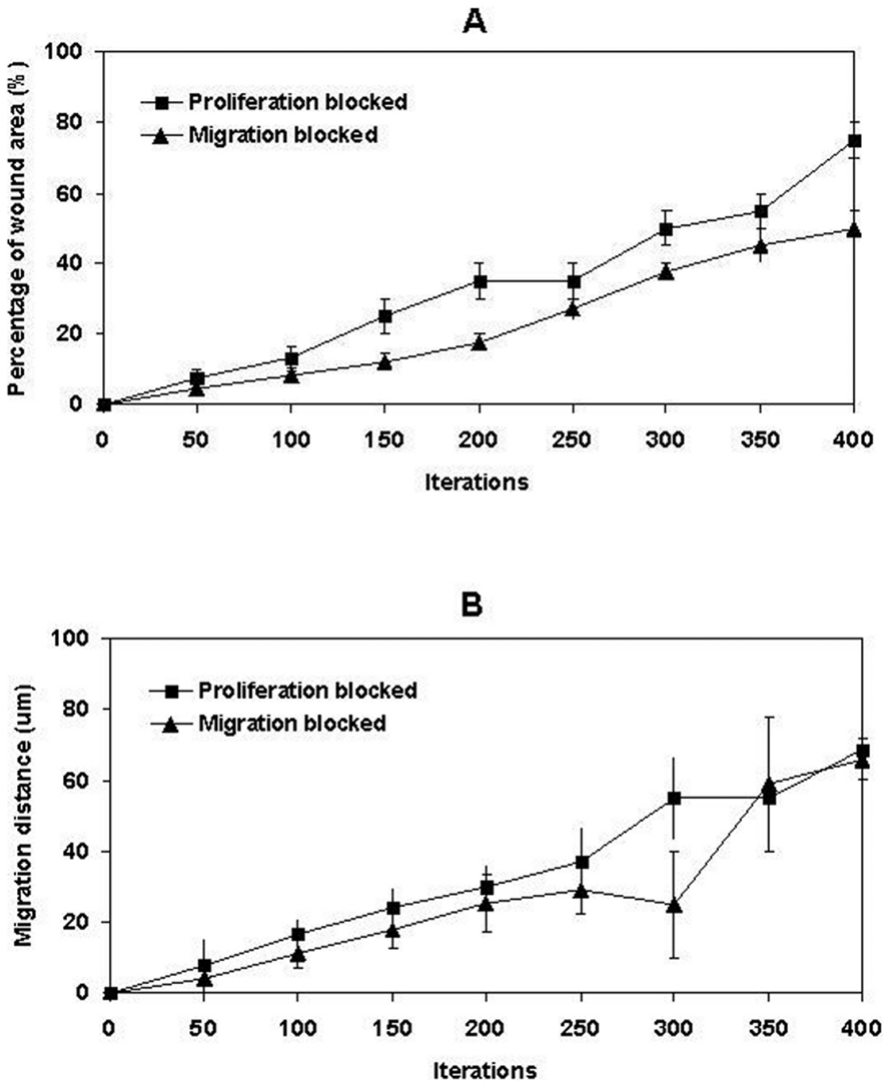
In virtuo investigation of the functions of TGF-β1 during epidermal wound healing. TGF-β1 was assumed to be responsible for 60% of cell migration and inhibit 60% of cell hyperproliferation. Its migration function and proliferation inhibition function at subcellular level were blocked respectively in two in-virtuo experiments and the virtual epidermal wound was then simulated 400 interactions. (A) Migration distance and (B) percentage of the wound area that covered by epidermal cells were used to evaluated the influences of the block of two functions on epidermal wound healing.

Based on this assumption, it was found that if its function on cell migration was blocked then wound healing was partially slowed down (as shown in Fig. 9A–C). If its inhibitory function on cell proliferation was blocked, then the wound healed in a relatively disorganised manner (Fig. 9D–F). Model analysis demonstrated that this was because the cells on the migration tongue also proliferated and there was only one single NHK population, which was both proliferative and motile. The influence of TGF-β1 on epidermal wound healing was also evaluated by measuring the percentage of the wound area that was covered by epidermal cells (Fig. 10A) and epidermal cell migration distance (Fig. 10B). These *in virtuo* experiments based on proposed quantitative contributions for TGF-β1 actions on cell migration and proliferation gave some interesting insights into how this regulatory factor may be acting under normal wound healing conditions.

## Discussion

Previous work from our group has used an agent based keratinocyte colony formation model, to improve our understanding of the influences of cell proliferation and migration on the ability of keratinocytes to heal a scratch wound in a Petri dish [**5**]. However in this model it was not possible to investigate the influence of any specific sub-cellular pathways on multi-cellular behaviors or tissue morphogenesis. Agent-based modeling approaches are ideal for reducing the complexity of biological systems by abstracting away the micro-level or sub-cellular details to produce a global view of the biological system. These allow the testing of hypotheses and the designing of new informative experiments. The regulation of epidermal homeostasis involves a complex interplay between different generic and genetic mechanisms; cells actively change their behaviors and properties as a consequence of internal decisions or ‘sub-cellular rules’ that are encoded in the genetic information. It would be very useful to investigate specific sub-cellular mechanisms in a model that can combine the description of a cell with a description of the “sub-cellular rules” that dictate the change of its behaviors or parameters [**2**], [4], [**63**].

The aim of this research was to develop an agent and COPASI based multi-scale model and go to the next level of complexity by combining the description of cell behaviors at the cellular level during epidermal wound healing with the description of TGF-β1 mechanisms (e.g expression, function etc) at the sub-cellular level.

To do this the previously developed agent based keratinocyte colony formation model [**5**] was first extended to an epidermal wound healing (re-epithelialisation) model by including new biological rules about the emergent responses of keratinocytes to various wound injury signals abstracted from an extensive published literature. At this stage the rules were still at the cellular level and only governed basic cellular behaviors such as cell proliferation, migration and differentiation. The sub-cellular details of TGF-β1 expression, signaling and regulation were then simulated using COPASI and integrated with the re-epithelialisation model. The model could now be used to simulate various cell-cell and cell-ECM interactions at the cellular level, but also to explicitly investigate the regulation of TGF-β1 on keratinocyte behaviours at the sub-cellular level.

The validity of the basic biological rules at the cellular level and the sub-cellular mechanisms used in the COPASI were tested by comparing the simulation results of both intact epidermis and the epidermal wound healing process with corresponding research reports. The results demonstrated that the model successfully simulated the reported behavior of keratinocytes and of TGF-β1 sub-cellular mechanisms during epidermal wound healing. Overall the initial validity testing of the model seemed promising and quickly led to the generation of hypotheses for exploration.

The model was first used to create a virtual epidermis from scratch, which was then virtually injured and used to analyse the proliferation and migration of keratinocytes during re-epithelisation. Our model demonstrated that during re-epithelialisation proliferative and migratory keratinocytes were located in different parts of the wound bed. Epithelial cell migration was speculated to be not dependent on cell proliferation [**10**], [**59**]. However, our model analysis indicated that cell proliferation is equally as important as cell migration (as other reports have suggested [**11**], [58], [**64**], since an extra source of keratinocytes is crucial in wound healing for the continuous migration of epidermal cells as a cohesive sheet.

The role of TGF-β1 in re-epithelialisation seems initially very contradictory [**8**], [11], [39], [41], [44], [49]–[51], [60]–[**61**]. This reflects an inadequate understanding of the mechanisms of TGF-β1 partly due to the limitations of many experimental systems or models [**8**], [49], [**65**]. For example, the traditional 2D *in vitro* wound healing models in tissue culture plates not only lack complete differentiation of the cultured epithelium [**58**], but also induce TGF-β1 synthesis due to the absence of any BM [**23**]. Although organotypic models have fully differentiated keratinocytes, they also have limitations as wound healing is affected both by tissue architecture and the participating cell types [**53**]. *In vivo* approaches, including transgenic mouse models, are able to embrace globally what happens during skin wound healing but the complexity provided by the coexistence of many cell types and many sub-cellular pathways makes it difficult to identify and address specific questions about TGF-β1 [**11**], [44], [48], [**66**].

In this research the integrated modelling approach was used to investigate the functions of TGF-β1 at both sub-cellular and cellular levels. Model analysis demonstrated that a temporal-spatial concept of TGF-β1 expression/signaling was crucial to understanding its roles in epidermal wound healing. As the model illustrated, only the NHKs close to the migration front were simulated to express TGF-β1 due to their interactions with the provisional matrix. It was this migratory keratinocyte population that was regulated by TGF-β1, while the proliferative keratinocyte population was not influenced by TGF-β1. Therefore, the biological roles of TGF-β1 on wound healing were not conflicting in the context of healing a wound in a 3D epithelial tissue.

Our model supports TGF-β1 playing an important role in keeping the balance between migration and proliferation for normal wound healing. Model analysis further indicated that any disruption of TGF-β1 expression or signalling could influence the healing process leading to chronic wounds or hypertrophic wounds as indicated by biological research [**66**].

Simulation of the sub-cellular mechanisms in the epidermal wound healing suggested that such biological information, such as to what extent TGF-β1 can influence cell migration and proliferation, is crucially important for us to calibrate the model before it can be used by biologists to explain or investigate more complex biological questions in the laboratory. The experience of developing this integrated model also clearly demonstrated the advantages and disadvantages of the simple biological rules in the agent based model and the mechanisms in the COPASI model. A balance between simple biological rules and complex sub-cellular mechanisms is crucially important to develop a multi-scale model based on current biological knowledge.

It is obvious that there are still many limitations in this multi-scale model. It does not go into details on which attachment factors or mitogens are produced during the wound healing process, or how cells respond to factors other than TGF-β1. However despite these limitations the current study demonstrates the potential of such models to become a powerful tool for understanding complex biological systems at a system level and guiding our research.

In summary, in this study we describe a novel computational model of epidermal wound healing, based on previously published experimental work both at the cellular and sub-cellular levels, enabling hypotheses to be readily generated and tested. This synergy between different modeling approaches has the potential to become a powerful tool for understanding how cells organize into tissues. An immediate “deliverable” to biologists is an easy to follow 3D *in virtuo* model which explains how TGF-β1 acts in wound healing in a highly coordinated manner to inhibit cell proliferation and stimulate cell migration. Further the model can now be used to investigate hypotheses behind chronic wound healing and scarring or to predict the number of keratinocytes with colony forming ability required to heal extensive skin wounds. At the sub-cellular level, the model can be employed for example to improve our understanding of how to deliver TGF-β1 to achieve better wound healing. The model can also be modified to explore questions about the application of other growth factors which biologists currently try to answer at an empirical level.

## Supporting Information

Movie S1 The integrated model is used to illustrate the healing process of a large wound.
(7.38 MB WMV)
